# Grainyhead-like 2 is required for morphological integrity of mouse embryonic stem cells and orderly formation of inner ear-like organoids

**DOI:** 10.3389/fcell.2023.1112069

**Published:** 2023-09-07

**Authors:** Blaise Forrester-Gauntlett, Linda Peters, Björn Oback

**Affiliations:** ^1^ Animal Biotech, AgResearch, Hamilton, New Zealand; ^2^ School of Science, University of Waikato, Hamilton, New Zealand; ^3^ School of Medical Sciences, University of Auckland, Auckland, New Zealand

**Keywords:** autosomal-dominant hearing loss, inner ear, embryonic stem cells, organoids, grainyhead, quantitative morphometry, cell adhesion, cadherin

## Abstract

Mutations in the transcription factor gene grainyhead-like 2 (*GRHL2*) are associated with progressive non-syndromic sensorineural deafness autosomal dominant type 28 (*DFNA28*) in humans. Since complete loss of *Grhl2* is lethal in mouse embryos, we studied its role during inner ear pathology and hearing loss *in vitro*. To this end, we generated different homozygous deletions to knockout *Grhl2* in mouse embryonic stem cells (*Grhl2-KO* ESCs), including some mimicking naturally occurring truncations in the dimerisation domain related to human *DFNA28*. Under naïve culture conditions, *Grhl2-KO* cells in suspension were more heterogenous in size and larger than wild-type controls. Adherent *Grhl2-KO* cells were also larger, with a less uniform shape, flattened, less circular morphology, forming loose monolayer colonies with poorly defined edges. These changes correlated with lower expression of epithelial cadherin *Cdh1* but no changes in tight junction markers (*Ocln, Tjp2*) or other *Grhl* isoforms (*Grhl1, Grhl3*). Clonogenicity from single cells, proliferation rates of cell populations and proliferation markers were reduced in *Grhl2-KO* ESCs. We next induced stepwise directed differentiation of *Grhl2-KO* ESCs along an otic pathway, giving rise to three-dimensional inner ear-like organoids (IELOs). Quantitative morphometry revealed that *Grhl2-KO* cells initially formed larger IELOs with a less compacted structure, more eccentric shape and increased surface area. These morphological changes persisted for up to one week. They were partially rescued by forced cell aggregation and fully restored by stably overexpressing exogenous *Grhl2* in *Grhl2-KO* ESCs, indicating that Grhl2 alters cell-cell interactions. On day 8, aggregates were transferred into minimal maturation medium to allow self-guided organogenesis for another two weeks. During this period, *Grhl2-KO* cells and wild-type controls developed similarly, expressing neural, neuronal and sensory hair cell markers, while maintaining their initial differences in size and shape. In summary, Grhl2 is required for morphological maintenance of ESCs and orderly formation of IELOs, consistent with an essential role in organising epithelial integrity during inner ear development. Our findings validate quantitative morphometry as a useful, non-invasive screening method for molecular phenotyping of candidate mutations during organoid development.

## Introduction

Hearing loss is the most common sensory disability, affecting over 5% of the world’s population and estimated to double by 2050 due to an ageing population (Exeter et al., 2015; WHO, 2023). Inherited dominant hearing loss accounts for approximately 40% of non-syndromic hearing loss and there are currently 68 genetic loci associated with the autosomal dominant form of hearing loss (Van Camp and Smith, 2023). Understanding the complex genetic basis of non-syndromic age-related hearing loss is essential for developing restorative or preventative measures but is complicated by genetic and clinical heterogeneity (Astuto et al., 2000; Keats, 2005). The auditory system requires 26 different cell types for functional hearing (Dror and Avraham, 2010) and 124 genes have been associated with non-syndromic hearing loss alone (Van Camp and Smith, 2023).

Grainyhead-like 2 (*GRHL2*) is associated with progressive, non-syndromic sensorineural deafness autosomal dominant type 28 (*DFNA28*) in humans. The gene encodes a highly conserved transcription factor (Bray and Kafatos, 1991) that contains an N-terminal transactivation domain, a unique CP2 DNA-binding domain and a C-terminal dimerisation domain (Ming et al., 2018). *DFNA28* was first identified in a multi-generational family with affected individuals displaying bilateral hearing loss. A single nucleotide polymorphism (SNP) in exon 13 (*c.1609dupC*), resulting in a frameshift and a premature stop codon in exon 14, was associated as the causative mutation (Peters et al., 2002). Variants in *GRHL2* rarely associate with autosomal dominant hearing loss, with only eight heterozygous, pathogenic variants known (Trebusak Podkrajsek et al., 2021). Half of these are located in the DNA-binding domain (Iwasa et al., 2016; Vona et al., 2013; Wu et al., 2020; Ji et al., 2014) and the other half in the dimerisation domain (Peters et al., 2002; Trebusak Podkrajsek et al., 2021; Iwasa et al., 2016); so far, none have been reported to affect the transactivation domain. No homozygous *GRHL2* mutations have been described in human *DFNA28*.

*GRHL2* is expressed exclusively in ectodermal and endodermal epithelia and their derivatives, suggesting a role in epithelial development, maintenance and repair (Auden et al., 2006; Werth et al., 2010; Senga et al., 2012; Mehrazarin et al., 2015; Walentin et al., 2015; Ray and Niswander, 2016). Homozygous *Grhl2* mutations in mouse are embryonic-lethal with defects in neural tube closure (Rifat et al., 2010). Consequently, analysing *Grhl2* function in inner ear development has been restricted to examining otocyst formation (Rifat et al., 2010) and *Grhl2* expression up to mid-gestation (Auden et al., 2006). Wild-type and heterozygous *Grhl2* KO mice show high expression of *Grhl2* in both the otic cup and otocyst at embryonic day 8.5–11.5. Subsequently, *Grhl2* expression is high in the vestibular and cochlear duct (Auden et al., 2006) as well as in the hair cells, support cells and stria vascularis (Peters et al., 2002). No neural tube defects were observed in heterozygous *Grhl2* KO mice which are viable, fertile and live a normal life-span (Rifat et al., 2010).

Apical junctional complexes are essential for epithelial remodelling in the developing inner ear (Bryant et al., 2002; Barald and Kelley, 2004). Formation of the otic vesicle involves tight junctions for determining cell polarity and adherens junctions for invaginating the placode to form the otic cup (Torres and Giraldez, 1998), as well as for maintaining sensory hair and non-sensory support cells in the sensory epithelium (Hibino and Kurachi, 2006; Nakajima, 2015). Loss of the tight junction protein claudin 14 (Cldn14) has been associated with autosomal recessive deafness *DFNB29* due to degeneration of the hair cells after birth (Wilcox et al., 2001; Ben-Yosef et al., 2003). Cdh1 regulates cell-cell adhesion in many epithelia, including the inner ear. Loss of Cdh1 in otic tissue impaired hearing function through failure to determine cell polarity (Desai et al., 2009) and establish a functional epithelial basal layer within the stria vascularis (Trowe et al., 2011).

During embryogenesis, GRHL2 activates genes involved in establishing functional epithelia via apical junction complexes (Werth et al., 2010; Walentin et al., 2015; Walentin et al., 2016). Specifically, Grhl2 binds to the *Cldn4* and *Cdh1* promoters to upregulate their expression, which is critical for forming tight and adherens junctions, respectively (Senga et al., 2012). In *Grhl2* KO mice, *Cldn4* and *Cdh1* were reduced and compensatory *Cdh2* mRNA expression was increased, allowing formation of intact adherens junctions (Werth et al., 2010). Loss of *Grhl2* dysregulated epithelial morphogenesis and impaired epithelial barrier function (Chen et al., 2010; Varma et al., 2012; Gao et al., 2013; Petrof et al., 2014; Hinze et al., 2018; Kersbergen et al., 2018).

Since *Grhl2* KO mice die at mid-gestation, cell culture models have been used to better define the *Grhl2* loss-of-function phenotype during ear development. Embryonic stem cells (ESCs) can give rise to all somatic cell types (“pluripotency”) and self-renew indefinitely from single cells (“clonogenicity”). This allows for long-term culture of ESC-derived three-dimensional (3D) cultures to assay *in vitro* differentiation potential of genetically engineered cells. Organoid differentiation from ESCs was initiated by forming floating embryoid bodies (EBs), derestricting cytomechanic forces, tissue movement and growth, to create miniaturised organ-like aggregates. These organoids offer an alternative *in vitro* model system, which mimics aspects of *in vivo* development, to study human disorders, and regenerative therapeutics (Artegiani and Clevers, 2018). This is especially relevant for modelling *GRHL2*, which is involved in cell-cell and cell-extracellular matrix (ECM) interactions during epithelial morphogenesis.

ESCs have been differentiated into inner ear-like organoids (IELOs) using the self-organising stem cell culture technique known as SFEBq (serum-free floating culture of EB-like aggregates with quick re-aggregation) (Eiraku et al., 2008). The resulting 3D-organoids resemble the saccules of the vestibular apparatus and recapitulate aspects of inner ear formation *in vitro* (Koehler et al., 2013; Koehler and Hashino, 2014; DeJonge et al., 2016; Liu et al., 2016; Nie et al., 2017; Tang et al., 2019). To elucidate the role of *Grhl2* during inner ear biogenesis, we engineered different homozygous deletions of *Grhl2* in ESCs. This included truncations in the three different functional domains of *Grhl2*, two of which are associated with human *DFNA28*. We demonstrate that all of these mutations cause pathological features in naïve ESCs and IELOs that are consistent with an essential role of *Grhl2* in organising epithelial integrity during inner ear development.

## Materials and methods

Chemicals were supplied by Sigma-Aldrich (Auckland, New Zealand), unless indicated otherwise.

### Generation of *Grhl2-KO* ESCs

Parental v6.5 ESCs were cultured on 0.1% gelatin-coated tissue culture dishes at 37°C in 2iL medium, comprising of PD0325901 (0.4 μM), CHIR99021 (3 μM), and recombinant human LIF (20 ng/mL) in Hepes-buffered DMEM (hDMEM) with N2 and mixed 1:1 with Neurobasal medium, supplemented with B27 and 1 mM L-glutamine (“N2B27”) (Ying et al., 2008). Cells were passaged using 0.25% trypsin.

For knocking out (KO) two or three functional domains of Ghrl2, wild-type (WT) ESCs were transfected with pools of two different *gRNA/Cas9* expression vectors from Santa Cruz Biotechnology (SCBT, #sc-434250), targeting Cas9 to exons 2 and 3 and co-expressing 2a-linked green fluorescent protein (GFP). Homology-directed repair (HDR) plasmids from a genome-scale CRISPR knock-out (GeCKO) v2 library (Sanjana et al., 2014) were used for inserting red fluorescent protein (RFP) and a 2a-linked puromycin resistance gene into exons 2 and 3 (#sc-434250 HDR). For targeting the dimerisation domain in exon 13, two gRNAs were cloned into the pSpCas9(BB) 2A puro (PX459) plasmid containing a puromycin antibiotic selection marker (Ran et al., 2013). Sequences for genome editors are shown in Supplementary Table S1. To isolate correctly targeted clones, cells were selected with 2 µg/mL puromycin 7 days after transfection with lipofectamine^®^ LTX. Resulting puromycin-resistant clones were picked approximately 10 days later and expanded in puromycin-free medium. Picked clones were analysed for correct targeting by endpoint polymerase chain reaction (PCR) of genomic DNA using the KAPA2G Fast HotStart PCR kit under standard conditions (Merck, New Zealand). Exons 2 and 3 of the non-targeted wild-type locus were detected with primer pairs 2 and 3, respectively. Successful targeting at exon 2 and 3 was detected with exon primers paired with forward or reverse primers targeting the transgene insert (F^HDR^ and R^HDR^). Integration of the *HDR-RFP_puro* and *gRNA/Cas9_GFP* plasmids was tested using primer pairs F^Plasmid^/R^HDR^ and F^Plasmid^/R^GFP^, respectively. Primer sequences are shown in Supplementary Table S2. Excised bands were verified by Sanger sequencing (Massey Genome Service, New Zealand).

### *Grhl-*overexpression cell lines

Full-length mouse *Ghrl1* and *Ghrl2* open reading frames were synthesised by GeneArt (ThermoFisher, New Zealand) and ligated into the EGFP N1 expression vector (Clontech) under control of the CMV promoter. Above described *Grhl2-KO* and wild-type ESC strains were transfected with lipofectamine LTX, placed under 1 mg/mL G418 antibiotic selection and GFP expression confirmed. Selected overexpression strains were subjected to genomic and RT-PCR to validate integration and expression of *Ghrl1* and *Ghrl2*, respectively, with appropriate primer pairs (Supplementary Table S2).

### IELO differentiation

All *Grhl2-KO* and overexpression ESC strains described above were subjected to IELO differentiation. Culture was carried out as described, including centrifugation at 200 × *g* for 3 min to promote EB-like formation via SFEBq-spin (Liu et al., 2016; Longworth-Mills et al., 2016), and Wnt signalling modulation through CHIR 99021 treatment on Day 8 (DeJonge et al., 2016). ESCs were cultured in ectodermal differentiation (ED) medium (95.5 mL GMEM [Gibco, ThermoFisher, #11710 035], 1.5 mL KO serum replacement [Gibco, ThermoFisher, #10828 010], 100 μL sodium pyruvate [1 M], 1 mL MEM-non-essential amino acids [100x], 180 μL β-mercaptoethanol). Cells were dissociated with trypsin, washed in PBS and GMEM and resuspended in 1 mL of ED medium. Single cell suspensions were passed through a 40 μm cell strainer (Corning, Sigma #352350), washed with ED medium, diluted to 3 × 10^4^ cell/mL and 100 µL (3,000 cells per well) seeded in ultra-low attachment 96-well plates overnight (Corning Costar^®^, Sigma CLS3474). On day 1, 2% (v/v) matrigel was added to promote formation of a basal membrane (Corning, Sigma #354230). On day 3, non-neural ectoderm was induced and mesendoderm formation inhibited with 10 ng/mL BMP4 (ProSpec, United States, #cyt 361) and 1 μM SB 431542 (Stemgent, United States, #04 0010 05), respectively. On day 4.5, pre-placodal ectoderm was induced with 25 ng/mL FGF2 (R&D systems, United States, #233 fb) and 1 μM LDN 193189 (Reagents Direct, United States, #36 f52). On day 8, aggregates were removed from their wells, pooled by cell strain and treatment, washed with PBS and resuspended in maturation medium (Advanced DMEM/F12, N2 supplement, 1% Matrigel) and 3 μM CHIR 99021. Single aggregates were plated in a 96-well low-attachment plate and incubated for 48 h. On day 10, aggregates transitioned to self-guided IELO culture in maturation medium with daily half medium changes for the next 10–20 days. For each experiment, 16 wells served as controls by removing either substrate (matrigel), cytokines (BMP4, FGF2) or small molecules (SB 431542, LDN 193819) from the ED medium. Additionally, 3,000 ESCs were cultured in 2iL to ensure normal growth prior to differentiation.

For complementation, KO- and WT-ESCs were prepared as described above for IELO differentiation (trypsinised, washed, size-selected) and seeded together, each at 1.5 × 10^4^ cell/mL per 96-well. Images of mixed colonies were acquired after 1 day in co-culture.

For EB-like formation in suspension, ESCs were trypsinised into single cells, washed, and seeded in 2iL medium at 1.7 × 10^5^ cells/cm^2^ per Petri dish. After 6 days in suspension culture, with occasional agitation, colonies were sequentially filtered through 70 μm and 100 μm cell strainers and collected in a Petri dish. Size-selected EB-like aggregates were placed into wells of a 96-well low-attachment plate with ED medium and incubated for 24 h before imaging.

### RNA and cDNA isolation

Cells were either processed fresh or snap-frozen in liquid nitrogen. Following lysis in *RNA*GEM™ Tissue *PLUS* (microGEM, Custom Science, New Zealand), cDNA was synthesised as described using Superscript IV (Bray and Kafatos, 1991). Reverse transcriptase was omitted in one sample, each time a batch was processed for cDNA synthesis (‘-RT’). Primers were designed using NCBI/Primer-BLAST, spanning introns, when possible (Supplementary Tables S2, S3), and synthesised by Integrated DNA Technologies (IDT, IA, United States).

### Reverse transcriptase quantitative PCR (RT-qPCR)

For RT-PCR and RT-qPCR, a Mic PCR instrument (BioMolecular Systems, United States) was used. All experiments were performed with the Takara Bio SYBR^®^ Ex Taq qPCR master mix (Norrie Biotech, New Zealand). The master mix consisted of 1.0 µL of each primer (10 µM), 10.0 µL master mix, 0.4 µL ROX dye, 7.44 µL DEPC water and 1.0 µL cDNA template. The following four-segment program was used: 1) denaturation (20 s at 95°C); 2) amplification and quantification (60 s at 95°C, 20 s at 60°C, followed by a single fluorescent measurement repeated 40 times); 3) melting curve (95°C for 5 min, final extension at 70°C for 1 min, heating at 0.003°C/s to 95°C while continuously measuring fluorescence); and 4) cooling to 4°C. Product identity was confirmed by gel electrophoresis and melting curve analysis. Assays were optimised to ensure i) a single melting peak corresponding to the correct PCR product size, ii) sufficient dynamic range over four orders of magnitude by running a 4-fold dilution series and iii) reduced or absent primer-dimer formation. Only the primer pairs that passed these quality controls were used for analysis. Relative quantification was carried out as described (Ruijter et al., 2009), taking reaction efficiency into account by amplification curve analysis. The mean reaction efficiencies for each assay are listed (Supplementary Table S3). When amplicons were not detected (as indicated by failure to amplify and/or absence of a single specific melting peak) an arbitrary baseline was set for that sample with a Ct of 35, which was normalised on the geometric mean of three or four reference genes.

### Alkaline phosphotase (AP) stain

AP activity was determined using a Leukocyte AP Kit (#86R), based on α-naphtholum coupled with diazonium salt, according to the manufacturer’s protocol. Cells were counterstained with Hoechst 33,342 and mounted in DAKO. Qualitative and quantitative comparisons were made using brightfield and fluorescence microscopy, respectively.

### Immunoblotting

Proteins were extracted with RIPA buffer (0.025 mL 1 M Tris/HCl pH 7.4, 0.3 mL 1 M NaCl, 0.05 mL 1 M EDTA, 0.5 mL 1 M Triton-X 100, 0.5 mL 10% SDS, 48 mL H_2_O and 1 pill cOmplete™ mini EDTA-free [Roche, New Zealand, #040693116001]). Protein extracts were separated by NuPage 4%–12% SDS PAGE, transferred onto a PVDF membrane (Amersham Life Science) and probed with anti-proliferating cell nuclear antigen (PCNA, Abcam, #ab29) and histone 3 (H3, Abcam, #ab1791) for 4°C overnight. Following incubation with a secondary antibody for 30 min at room temperature, bands were visualised with enhanced chemiluminescence. Signal intensity was normalised for the H3 signal and quantified using a Bio Rad image Quant Analyser and Image Lab 6.0 (Bio-Rad Laboratories Inc., New Zealand) (Supplementary Tables S4, S5). After imaging, the blot was stained with Ponceau, rinsed briefly in tap water to remove excess stain and place into a zip lock bag to image on the photocopier. For re-probing, the blot was stripped using 20 mM TCEP in 1% SDS solution for 2 h at 55°C and washed thoroughly in tap water before proceeding with washes, blocking and antibody incubation as above.

### Cryosections

IELOs were fixed in 4% (w/v) paraformaldehyde/4% (w/v) sucrose at room temperature for 20 min (Day 1–10 samples) or 1 h (Day 11–30 samples), washed in PBS and incubated in increasing concentrations of sucrose (10, 20, 30%) until they sank to the bottom. IELOs were embedded in Tissue-Tek O.C.T. Compound, frozen in an isopentane/dry ice slurry and stored at −80°C. Serial 7 μm sections were cut on a Leica CM1850 UV cryostat at −14°C and collected on Polysine^®^ slides, coated in 2 g/L gelatin.

### Immunofluorescence (IF)

Fixed sections were quenched in NH_4_Cl for 10 min, permeabilised in 0.1% (v/v) Triton X-100 in PBS for 10 min at room temperature, blocked in 5% goat or donkey serum for at least 1 h and incubated with the primary antibody at 4°C overnight. Samples were rinsed in PBS and incubated with the secondary antibody and 5 µg/mL Hoechst stain, diluted in blocking solution, for 30 min at 37°C (Supplementary Tables S4, S5). Samples were washed thrice in PBS and once in H_2_O before mounting in Dako (Dako, Australia) and sealed with nail polish. Images were taken on an epifluorescence microscope (AX-70, Olympus, New Zealand) equipped with a Spot RT-KE slider CCD camera (Diagnostics Instruments Inc., Sterling Heights, MI, United States) and analysed with ImageJ 1.45S.

### Quantitative morphometry

For ESC morphometry, single cells and group cultures were seeded onto flat-bottomed 96-well plates and 8-well chambers, respectively, with images taken every day (AMG, EVOS FL digital microscope, United States). Clonogenicity was defined as the proportion of colonies formed per single cells seeded after 12 days. For all other measures, cells were fixed in 4% paraformaldehyde for 15 min at room temperature, stained with Eosin/Hoechst 33,342 and images acquired under transmission or fluorescence settings. Images were loaded into OrganoSeg software (Borten et al., 2018) and cells or colonies segmented to produce binary images. Binary images, along with the raw brightfield and fluorescent images, were imported into CellProfiler 3.1.5 software (McQuin et al., 2018) to create masks for cells, colonies and backgrounds of each image type. The mean background intensity was subtracted from each corresponding image for normalisation before measuring area, cell density and circularity. All masked images, raw and calculated measurements were exported as a .csv file and analysed in R Studio (R Core Team, 2023). Each experimental run was treated as a technical replicate with cell strains (clonogenicity, area) or clonal colonies (density, circularity) as biological replicates. Nuclei were manually counted in ImageJ ((Schneider et al., 2012) Version: 1.4.3.67). For IELOs, images were taken daily for 9 days after seeding and processed as for naïve ESCs using OrganoSeg and CellProfiler software to measure area, perimeter, circularity and compactness. Each experimental run was treated as a technical replicate with clonal colonies as biological replicates.

### Cell proliferation assays

Proliferation rate was measured from clonal colonies and group cultures, seeding either single cells onto 96-well plates or 1.2 × 10^4^ cells/well onto 12-well plates, respectively. After 5 days of culture, all cells were lifted from each well, dispersed with trypsin, and single cells counted with a haemocytometer to determine the population doubling (PD) time.

$$Population\;doubling\;time\;\left(h\right)=\frac{\ln\left(2\right)\left(\frac{N_{0}}{N_{T}}\right)}{∆time}$$

Real-time changes in cell number, viability, and morphology were quantified as a cell index (CI) using an RTCA-SP xCELLigence^™^ system (Roche, New Zealand). Cells were seeded in 100 μL 2iL^+2% FBS^ medium at 3,000 or 5,000 cells per well onto 0.1% gelatin-coated 96-well E-plates (ACEA Biosciences, New Zealand, #5232368001). Each ESC strain (N = 6, N = 4 and N = 3 for WT, 2KO/3KO*,* and 1KO strains, respectively) and negative control (medium only) was run in 6-8 technical replicates per run. CI readings were taken every 15 min for 12 days. For RTCA data analysis, normalised CI values were calculated for each well by normalising the CI value 30 min after media changes. Normalised CI values were automatically calculated by RTCA Software 2.0 (Roche) and used threshold data for baseline corrections. Once normalised and baseline-corrected, a sigmoid curve was fitted using the Growthcurver package in R (Sprouffske and Wagner, 2016). Outlier wells were removed before processing.

### Statistical analysis

Variables were analysed using ANOVA (aov in R) by genotype or experimental conditions (R Core Team, 2023). Means were compared using TukeyHSD (multcompLetters4 in R). Values are presented as box-whisker plots, including individual data points, mean and 95% confidence interval for the median, unless indicated otherwise. For RT-qPCR gene expression analysis, log transformed relative expression was analysed by linear mixed-effects model fit by REML (Pinheiro et al., 2007). A Ct value of 35 was given for missing values for computation of the baseline relative expression.

## Results

### Targeted disruption of *Grhl2*

**FIGURE 1:**
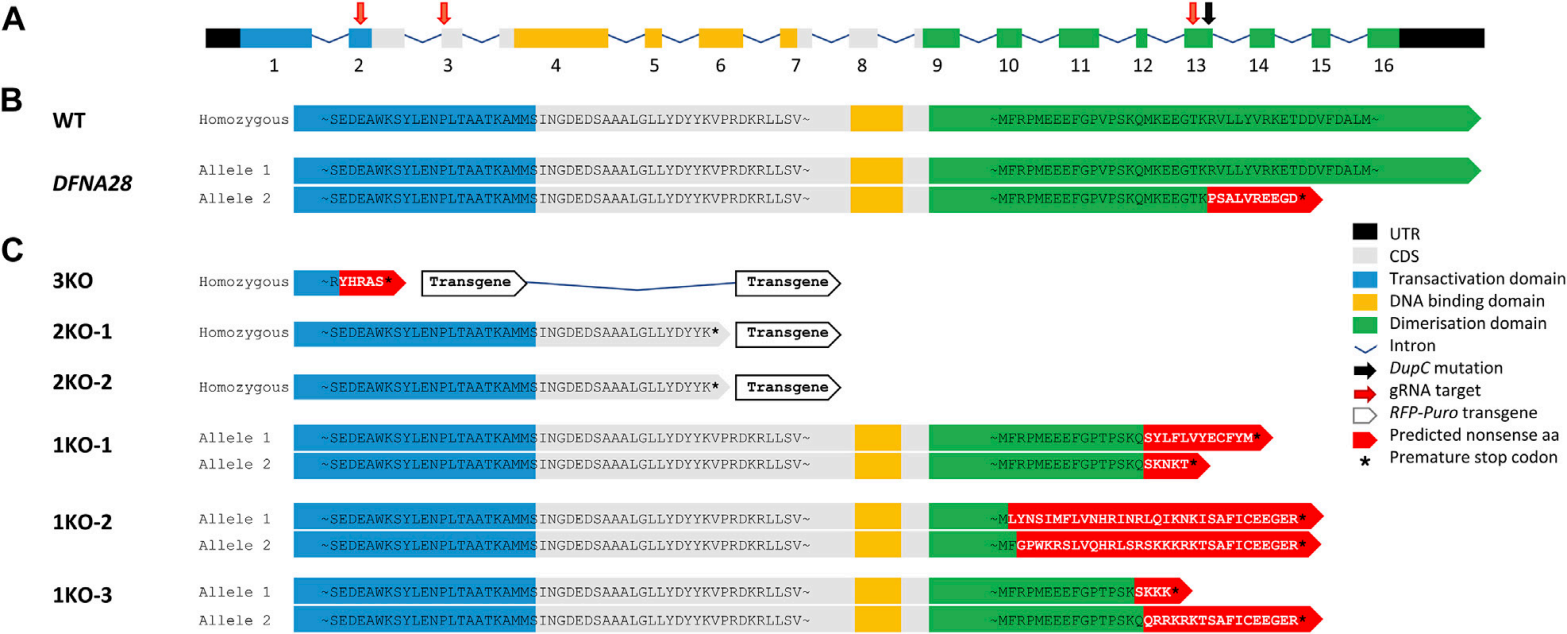
*gRNA/Cas9* genome editing of murine Grhl2. **(A)** Murine *Grhl2* wild-type (WT) gene structure with untranslated regions (black), numbered exons of coding sequence (CDS, grey), including functional domains for transactivation (blue), DNA-binding (yellow) and dimerisation (green). Mapped to the sequence are the *gRNA* targets (red arrows) and the *c.1609dupC* mutation in human *DFNA28* (black arrow). **(B)** Predicted amino acid (aa) sequences for murine WT and human *DFNA28* heterozygous genotype. **(C)** Predicted amino acid sequences for gene edited ESC strains with loss of three or two functional domains after homology-directed insertion of red fluorescent protein (*RFP*) and puromycin (*Puro*) transgene (3KO or 2KO-1, -2, respectively). Compound heterozygous *DFNA28-like* frameshift mutations after non-homologous end joining only affect the dimerisation domain (1KO-1, -2, -3).

ESCs were transfected with pools of three different *gRNA/Cas9* expression vectors and HDR plasmids, either targeting insertion of *RFP-puromycin* into exons 2 and 3 of the murine wild-type *Grhl2* gene or introducing indels into exon 13 (Figure 1A). These edits were designed to either create a complete KO by disrupting at least two functional protein domains or mimicking the SNP associated with human *DFNA28* by only targeting the putative dimerisation domain (Figure 1B). We identified 16 RFP-positive, puromycin-resistant clones with targeted HDR events. These included biallelic cell strains with loss of all three functional domains (3KO) or homozygous disruption of the DNA-binding and dimerisation domains (2KO-1, -2) (Figure 1C). Following NHEJ-mediated editing, we isolated three biallelic ESC strains with random deletions that introduced a premature stop codon in exon 13, similar to the human SNP. These *DFNA28-like* (1KO) strains (1KO-1, -2, -3) carried compound heterozygous frameshift mutations disrupting the putative dimerisation domain and were selected for further characterisation (Figure 1C). Wild-type and targeted ESC strains were screened for integration of the *HDR-RFP_puro* and *gRNA/Cas9_GFP* plasmids using primer pairs F^Plasmid^/R^HDR^ and F^Plasmid^/R^GFP^, respectively, and strains carrying plasmid integration were excluded (Supplementary Figure S1A, B). Disruption of wild-type exons 2 and/or 3 via HDR-mediated transgene integration was verified by genomic PCR and sequencing of the excised bands (Supplementary Figures S1C–E). Disruption of exon 13 via NHEJ-mediated indels was confirmed by Sanger sequencing (Supplementary Figure S1F). ESC strains were also verified using PCR to detect the presence of wild-type length mRNA transcripts (Supplementary Figure S1G). For 2KO and 3KO strains, the HDR transgene insert included an SV40 polyA transcription termination sequence leading to premature transcriptional arrest of mRNA resulting in the failure to amplify with mRNA primers spanning either exons 2-3 or exons 10-16. For 1KO strains, agarose gel electrophoresis was not able to resolve 1-4 bp differences introduced by NHEJ-mediated indels.

### Loss of *Grhl2* alters ESC morphology and disrupts colony integrity

**FIGURE 2:**
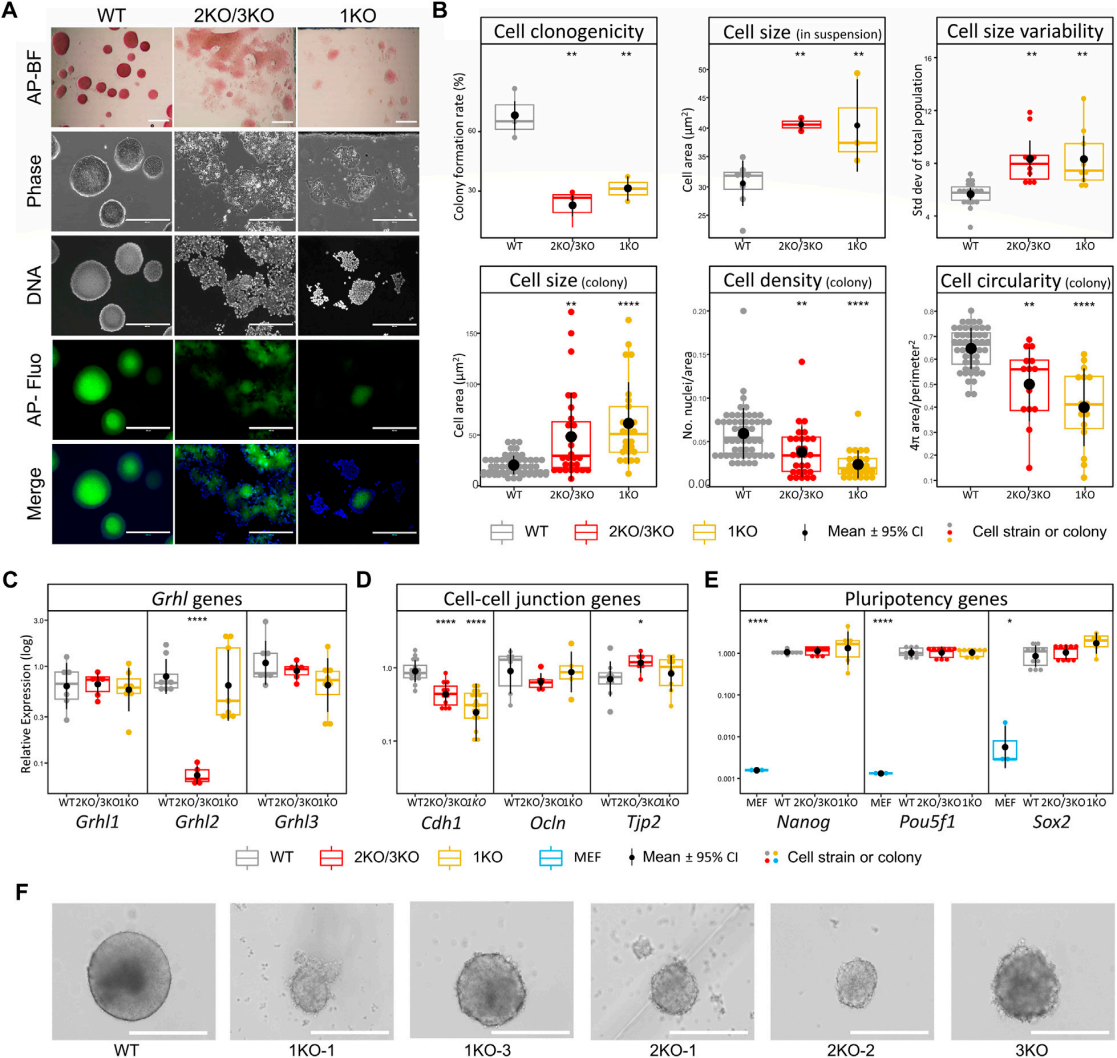
Loss of Grhl2 alters ESC morphology. **(A)** Qualitative morphology of representative *Grhl2-KO* grown in group cultures, compared to wild-type (WT) ESCs. Alkaline phosphatase (AP) activity was stained with a colorimetric assay and visualised with brightfield (AP-BF), phase contrast (Phase) or epifluorescence in the FITC channel (AP-Fluo). DNA was visualised with Hoechst 33,342. Scale bar = 400 µm. **(B)** Quantitative morphometry of single ESCs and ESC colonies. Colony formation assay from single cells (clonogenicity) was determined in several strains for each genotype (N = 3, N = 3 and N = 2 for WT, 2KO/3KO and 1KO, respectively). Single cells were sized in suspension with several strains analysed for each genotype (N = 6, N = 4 and N = 3 for WT, 2KO/3KO and 1KO, respectively). To determine cell size variability, each strain was analysed in 3 replicates (N = 1,122, N = 708 and N = 568 cells, respectively). Colony morphology and composition (cell size within colonies, density and circularity) was calculated using colonies from the colony formation assay (N = 49, N = 14, N = 13 colonies for WT, 2KO/3KO and 1KO, respectively). All measurements were taken 12 days after plating single ESCs. **(C–E)** Gene expression changes for *Grhl* transcription factors **(C)**, cell-cell junctional markers **(D)** and pluripotency markers **(E)**. Expression was normalised on the geometric mean of three reference genes. *, **** = *p* < 0.05, *p* < 0.0001, respectively. **(F)** Representative images of EB-like aggregates formed during hanging drop differentiation for several days. Scale bar = 400 µm.

We first evaluated the effect of *Grhl2* disruption on ESC morphology under naïve culture conditions. Qualitatively, adherent *Grhl2-KO* ESC colonies were flatter than the uniformly dome-shaped wild-type controls, instead forming heterogenous, loose monolayers with poorly defined edges, which showed alkaline phosphatase activity (Figure 2A). *Grhl2-KO* ESCs were less than half as clonogenic as wild-type controls (Figure 2B), whose colony formation rate was within the normal range for ESC strains in 2iL medium (Carey et al., 2015; Mulas et al., 2019). Quantitative morphometry showed that individual *Grhl2-KO* ESCs in suspension were 1.3-fold larger on average, but more variable, while their size in colonies was about 2.6-fold larger. They formed less compact colonies with a lower cell packaging density and reduced circularity (Figure 2B).

*Grhl2 e*xpression was barely detectable in 2KO/3KO cell strains, but not affected in 1KO strains, consistent with the endpoint PCR results reported above. Closely related *Grhl1* and *Grhl3* transcripts were not significantly altered (Figure 2C). Given the observed morphological changes, we investigated the expression of genes encoding candidate junctional proteins (Figure 2D). Epithelial cadherin (*Cdh1*) expression was 2-3-fold decreased in *Grhl2-KO* strains compared to wild-type, while *Ocln* and *Tjp2* expression, both encoding tight junction-associated proteins, showed small or no changes. To determine the state of ESCs, gene expression of canonical pluripotency markers was carried out (*Nanog*, *Sox2* and *Pou5f1).* Expression of these markers was compared between wild-type and edited ESC strains (3KO, 2KO-1, -2, and 1KO-1, -2, -3) and the data pooled for each knockout (3KO*/*2KO and 1KO). The results show abundant expression of pluripotency markers, with no significant differences between non-edited vs edited ESCs (Figure 2E). Further, morphologically normal KO-aggregates were formed after prolonged ESC culture in suspension and subsequent differentiation into EB-like structures (Figure 2F). Collectively, this indicates that *Grhl2* editing did not affect ESC differentiation *per se*.

### Loss of *Grhl2* reduces proliferation

We next determined the proliferation behaviour of *Grhl2-KO* ESCs. Based on cell counts of group cultures, population doubling times in wild-type ESCs was about 18 h, close to the range of the expected generation time (10–16 h) for naïve ESCs grown in 2iL medium (Carey et al., 2015). This was nearly doubled in both single and group cultures of *Grhl2–KO* ESCs with a mean generation time of 32 and 30 h, respectively (Supplementary Figure S2A). Using xCELLigence real-time kinetic profiling, both *Grhl2-KO* ESC genotypes behaved very similar, showing a flattened growth curve with lower peak and endpoint cell indices than wild-type controls (*n* = 4 biological replicates, Supplementary Figure S2B). Their growth rate, based on the RTCA cell index changes in group culture, was significantly reduced against wild-type cells (Supplementary Figure S2C). Reduced proliferation rates correlated with a 2.4-fold reduced abundance of PCNA, a cofactor of DNA polymerase and marker of cells synthesising DNA (Supplementary Figures S2D, E).

### Loss of *Grhl2* compromises early IELO differentiation

**FIGURE 3:**
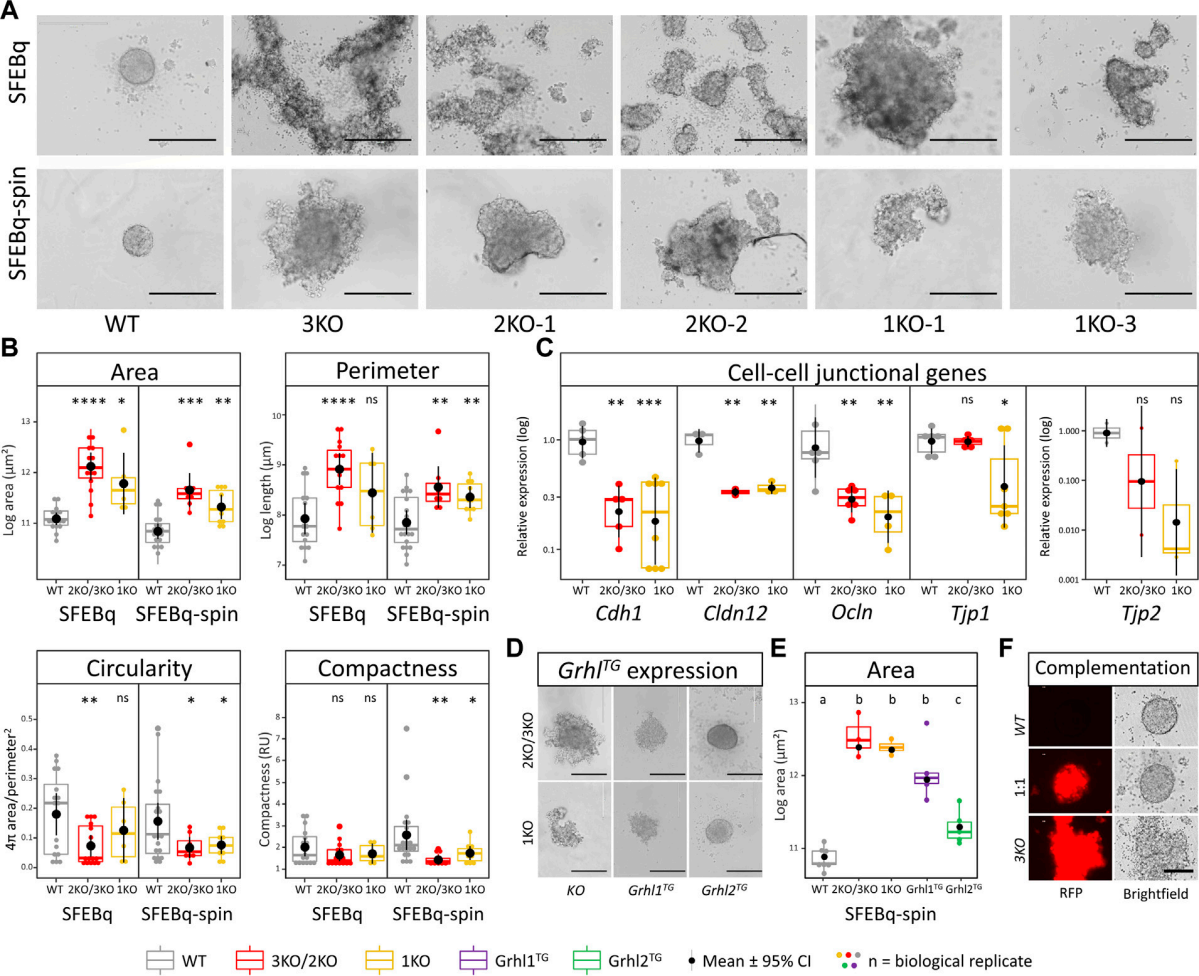
Loss of Grhl2 disrupts early IELO integrity. **(A)** SFEBq-spin partially restores IELO morphology; scale bar = 400 μm **(B)** Quantitative morphometry of *Grhl2-KO* IELOs. Morphology (area, perimeter, circularity and compactness) was determined after 1 day in culture. ns = non-significant. *, **, *** = *p* < 0.05, *p* < 0.01 and *p* < 0.001, respectively. **(C)** Gene expression changes for cell-cell junctional markers, normalised on the geometric mean of four reference genes. **(D)** Qualitative and **(E)** quantitative restoration of area by overexpressing *Grhl1,* or *-2* transgenes (*Grhl-TG*). ab = letters differ by *p* < 0.05; scale bar = 400 μm. **(F)** Complementation of 3KO-ESCs carrying a red fluorescent protein (RFP) transgene with WT-ESC (1:1). Non-aggregated 3KO and WT ESCs provide negative and positive controls, respectively. Scale bars = 200 µm.

To elucidate the role of *Grhl2* during differentiation, we generated 3D-organoids using the SFEBq technique. On day 1 after seeding, wild-type ESCs aggregated into uniform, spherical, tightly packed aggregates with a smooth surface. By contrast, both 2KO/3KO and *DFNA28L-KO* ESCs formed multiple heterogeneous, disorganised aggregates (Figure 3A). An additional centrifugation step (“SFEBq-spin”) partially rescued the KO phenotype and reduced the number of aggregates (Figure 3A). Quantitative morphometry showed that *Grhl2-KO* ESCs had a larger surface area and perimeter, were more eccentrically shaped and had a less compact structure (Figure 3B). These differences were still visible, but not as pronounced, in the SFEBq-spin group, indicating that centrifugation could partially rescue *Grhl2*-dependent cell-to-cell interactions during initial EB-like formation. The centrifugation resulted in smaller aggregates for both wild-type vs mutated ESCs and a reduced surface area in *Grhl2*-*KO* cells. KO aggregates had a more homogenous shape, as well as producing a single aggregate more often than smaller, discrete aggregates that very loosely clumped together. Although the KO EB-like morphology was improved by centrifugation (SFEBq-spin group), differences between wild-type and KO remained significant for area, perimeter, circularity and compactness, compared to SFEBq alone. Accordingly, *Cdh1* expression was about 4-fold decreased in *Grhl2-KO* strains compared to wild-type. By contrast to naïve ESCs, abundance of tight junction-related genes *Cldn12*, *Ocln*, and *Tjp2* was also about 4-fold reduced (Figure 3C).

EB-like formation by SFEBq-spin was also carried out with KO ESCs that carried randomly integrated *Grhl1* or *Grhl2* overexpression constructs. This resulted in a qualitative rescue of the phenotype for *Grhl2*, but not *Grhl1,* overexpressing lines, producing spherical and tightly packed colonies with a smooth surface (Figure 3D). Consequently, the surface area of *Grhl2*, but not *Grhl1*, stably overexpressing lines differed significantly from their parental KO but not from wild-type EB-like aggregates (Figure 3E). Rescue of morphology was also achieved by complementing RFP-positive *Grhl2-KO* ESCs with equal numbers of wild-type ESCs, indicating non-cell-autonomous actions of *GRHL2* (Figure 3F). All transgenic overexpression strains were verified by genomic PCR (Supplementary Figure S3A) and Sanger sequencing of the excised bands (Supplementary Figure S3B).

### *Grhl2* is not required for intermediate and late-stage IELO differentiation

Development of ESC-derived IELOs is defined by the addition of different small molecules and cytokines that initiate formation of defined developmental milestones. This process was tracked over time by imaging aggregates daily for up to three weeks. The gradual size increase was common for both genotypes and no qualitative differences in morphology were seen between the two methods used to form EB-like aggregates (Supplementary Figure S4A). Size and shape variations seen in the KO lines using SFEBq methods, carried through to subsequent days of intermediate differentiation and produced larger, less circular and less compacted aggregates (Supplementary Figure S4B).

**FIGURE 4:**
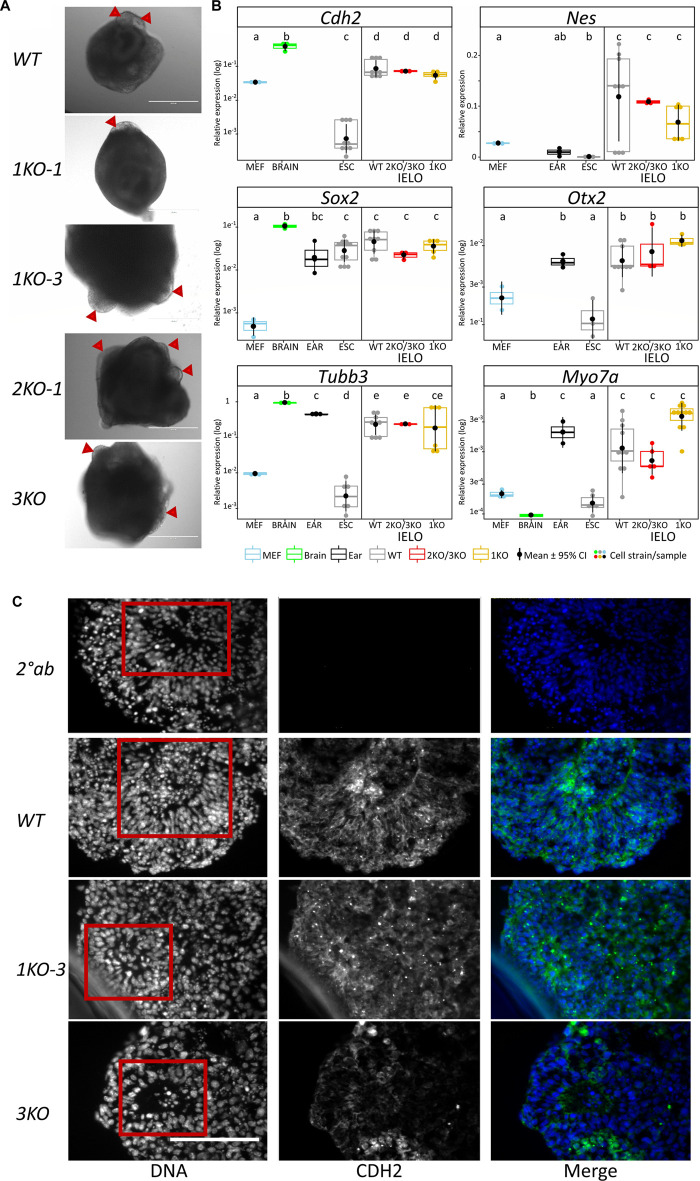
Grhl2 is not required for late IELO differentiation. **(A)** Brightfield images of IELOs showing presumptive otic vesicles on day 17–21 (red triangles). **(B)** Gene expression changes for neural (*Cdh2, Nes, Otx2, Sox2*), neuronal (*Tubb3*) and hair cell specific (*Myo7a*) markers in day 26 IELOs. Gene expression relative to *Gapdh*. abcde = samples with different letters differ by *p* < 0.05. **(C)** IELOs at day 21 contained neural rosettes (red box) with cells expressing CDH2 (green). Negative control was only incubated with the secondary antibody (*2° Ab*). DNA was counterstained with Hoechst 33,342 (blue). Scale bars = 400 µm.

On day 8, aggregates were transferred into minimal maturation medium to allow self-guided organogenesis for another two weeks. *Grhl2* was not required for late IELO differentiation as both KO- and WT-derived IELOs developed presumptive otic vesicles after day 17 (Figure 4A).

To assess differentiation, we quantified gene expression of neural markers (*Cdh2, Nes*, *Otx2, Sox2*), neuronal *Tubb3*, and hair cell-specific *Myo7a* in edited vs wild-type IELOs after 26 days in culture (Figure 4B). ESCs, MEFs and mouse adult ear or brain tissue served as negative and positive controls, respectively. There was significant expression, relative to undifferentiated ESCs and/or MEFs, of *Cdh2, Nes, Otx2, Sox2* and neuronal *Tubb3* in all D26 IELOs, indicating neural differentiation. Sensory hair marker *Myo7a* was equally expressed in both WT- and KO-genotypes and not significantly different from ear tissue. Protein expression of Cdh2 was present in both WT- and KO-IELOs, confirming neural differentiation around distinct rosette structures (Figure 4C). In summary, disruption of *Grhl2*, either by completely knocking out most functional domains or by mimicking the *DFNA28* mutation, altered early IEOL differentiation in ways that are consistent with Grhl2 organising epithelial integrity during development, but did not affect their ability to give rise to end-point IELO containing neuronal and sensory cells.

## Discussion

We have shown that mutagenising three, two or one of the *Grhl2* functional domains, including one truncation in the dimerisation domain associated with human *DFNA28*, causes loss of epithelial integrity in naïve ESCs and early IELOs. These findings are consistent with an essential role of *GRHL2* during inner ear development and validate quantitative morphometry as a non-invasive system for screening phenotypes of candidate pathogenic mutations in organoids.

### Mimicking pathogenic *Grhl2* mutations *in vitro*

All homozygous *Grhl2-KO* mice are embryonic-lethal, despite encompassing different mutations and genetic backgrounds, and none have reported a hearing loss phenotype (Werth et al., 2010; Menke et al., 2015; Walentin et al., 2015). Many inbred mouse strains display impaired hearing (Zheng et al., 1999; Noben-Trauth et al., 2003; Noben-Trauth and Johnson, 2009), including those from which the v6.5 ESC line derives (F1-C57BL/6 × 129/Sv). As both parental strains carry *Cdh23* mutations, which are associated with age-related hearing loss (Noben-Trauth et al., 2003), we used isogenic wild-type ESC controls to account for any potential phenotype caused by the background genetics. The *Grhl2* gene was disrupted in exons 2 or 3 by HDR-mediated transgene insertion or in exon 13 by NHEJ-mediated introduction of premature stop codons. The latter strategy was devised after an HDR-mediated approach failed to introduce the *dupC* mutation associated with *DFNA28*, possibly because the HDR template was complementary to the C57BL/6 reference sequence, which differs from the corresponding 129/Sv sequence by two SNPs and a 3 bp deletion. The NHEJ-introduced indels were not an exact match for the *c.1609dupC* mutation, resulting in 10 non-sense codons and a premature stop codon in exon 14, but caused a similar C-terminal truncation in the dimerisation domain. We also did not succeed in creating heterozygous *DFNA28-like* mutations. In the future, the sequence variations in exon 13 between C57BL/6 and 129/Sv could be exploited to introduce the +1 frameshift mutation to only one copy of the gene. Ideally, the human *GRHL2* sequence could be knocked into the mouse locus, before introducing any of the exact heterozygous pathogenic variants, either in the DNA-binding (Vona et al., 2013; Ji et al., 2014; Iwasa et al., 2016; Wu et al., 2020) or dimerisation domain (Peters et al., 2002; Iwasa et al., 2016; Trebusak Podkrajsek et al., 2021).

### Decreased cell proliferation in *Grhl2-KO* ESCs

In keratinocytes and cancer cell lines, *GRHL2* expression levels correlated with increased and decreased PCNA expression after overexpression and knockdown (Coronado et al., 2013), respectively. This is consistent with our results and with Grh proteins binding the PCNA promoter and enhancing expression (Hayashi et al., 1999). Proliferation promotes pluripotency reprogramming, whereas cell cycle arrest inhibits it and induces differentiation (Araki et al., 2010; Smith et al., 2010; Ruiz et al., 2011). Even though proliferation was reduced in *Grhl2-KO* compared to naïve ESCs, there were no signs of premature differentiation. It remains to be shown whether the anti-proliferative effect of *Grhl2* in ESCs was due to lengthening or blocking individual cell cycle phases, decreasing the proliferative cell fraction, inducing cell death or indirect effects. The assays we used, cell counts and RTCA, are both effected by the strength of cell-ECM or cell-cell interactions. Loss of *Grhl2* might weaken these interactions, increasing cell loss during media changes and overestimating the anti-proliferative effect. By contrast, wild-type ESCs grow as multi-layered colonies with a relatively small substrate footprint compared to the larger *Grhl2-KO* ESC monolayers, which will likely reduce their cell index. Uncoupling *Grhl2* effects on proliferation from substrate or cell adhesion will require different methods to measure self-renewal, such as flow cytometry of cells in suspension.

### *Grhl2*-deficient ESCs lose morphological features of naïve pluripotency

Pluripotency progresses from an early naïve to a late primed phase, represented by ESCs and epiblast-derived stem cells (EpiSCs), respectively (Nichols and Smith, 2009). ESCs form compact, dome-shaped 3D colonies, while EpiSCs form flattened 2D colonies and epiblast-like cells (EpiLCs) form a transitory intermediate with a heterogenous population of cells that are enriched in formative phase cells (Kalkan et al., 2017). Adherent ESCs exiting the naïve ground state show early morphological changes, involving cell movement and flattening, as they develop apicobasal polarity and epithelialize (Hayashi et al., 2011; Kinoshita et al., 2021). These *in vitro* events precede lineage priming and mirror cell biological changes *in vivo* when epithelial integrity consolidates during development from the pre- to the post-implantation epiblast (Smith, 2017).

*Cdh1* is essential for maintaining tightly packed colonies through cell-cell interactions. In EpiLCs or differentiating cells, *Cdh1* is downregulated or delocalised and cell-ECM interactions are increased. This results in a flattened elongated morphology, reduced proliferation and reduced clonogenicity. Loss or reduction of *Cdh1* pushes ESCs towards a flattened EpiLC morphology and impairs differentiation (Bao et al., 2009; Tian et al., 2011; Murray et al., 2013). Mimicking this phenotype, loss of *Cdh1*, ESC morphology, proliferation and self-renewal was also observed in *Grhl2-KO* cells.

*Grhl2*-KO ESCs were independently generated by mutagenesis of exon 2 (Chen et al., 2018). The authors observed that during the transition from wild-type ESCs to EpiLCs, *Grhl2* expression increased ten-fold, activating enhancers that maintained expression of genes involved in epithelialisation and cell adhesion (Chen et al., 2018). *Grhl2*-KO EpiLCs no longer formed compact colonies, increased mesenchymal *Cdh2* and decreased epithelial markers (*Cdh1*, *Tjp2*). This phenotype is reminiscent of *Grhl2-KO* IELOs. In contrast to our results, which documented larger, more heterogenous ESCs and flatter colonies, this phenotypic change was only described in EpiLCs. These differences may relate to lower levels of *Grhl2* expression, due to variations in culture conditions and pluripotency status. Both studies used the parental *v6.5* ESCs and 2iL supplementation, which results in a homogenous population shielded from differentiation (Tamm et al., 2013). However, our culture did not include 15% KO serum, which can introduce variable cell morphology, alter gene expression and lower chimaerism rates, all indicative of increased differentiation (Tamm et al., 2013; Martin Gonzalez et al., 2016). We also included N2/B27 supplemented Neurobasal/hDMEM, rather than KO DMEM base medium. The culture substrate differed between both studies, either comprising inactivated mouse embryonic fibroblasts (MEFs) or gelatin, which can impact colony morphology. Naïve ESCs reduce cell-ECM interactions by downregulating focal adhesion and integrins, forming tightly packed, rounded colonies on gelatin-coated plates (Taleahmad et al., 2017). Such weakly adherent substrate can maintain self-renewal and pluripotency without LIF or serum (Murray et al., 2013). By contrast, strongly adhesive surfaces (MEFs, fibronectin, laminin or collagen) can promote attachment and downregulation of pluripotency markers (Murray et al., 2013; Taleahmad et al., 2017). The pluripotent state is a continuum and these differences may collectively contribute to the observed differences in *Grhl2-KO* phenotype.

The effect in ESCs fits with the role of *GRHL2* during epithelial-to-mesenchymal transitions (EMT) and the reverse process (MET) where it also suppresses mesenchymal proteins (Chung et al., 2019). During EMT, cell-cell adhesion and polarity are lost as cells delaminate and migrate, while MET decreases cell motility, increases polarity and increases cell-cell interactions, leading to a more mesenchymal phenotype. *GRHL2* regulates EMT/MET plasticity through epigenetic methylation of CpG sites and nucleosome remodelling (Cieply et al., 2013; Frisch et al., 2017; Chen et al., 2018). A key event here is again the loss or reduction of *CDH1* expression, which weakens cell-cell contacts.

To better understand the effect of *Grhl2* KO on pluripotency will require a more in-depth analysis of the molecular and functional features that are diagnostic of naïve vs formative vs primed stem cells. This would require comprehensive profiling of their karyotype, transcriptional, epigenetic, metabolic and cell signalling properties, as well as their ability to form germ cells and pre- or post-implantation chimaeras, ideally at the single cell level (Kalkan et al., 2017).

### Compromised EB-like formation during IEO differentiation

The initial differentiation steps are critical for understanding the end-point in organoid production systems, which is often less reproducible (Huch et al., 2017). We observed reproducible changes during EB-like formation, the first step of generating IELOs. Under SFEBq conditions, *Grhl2-KO* ESCs formed large, loose, heterogenous aggregates. Brief centrifugation partially rescued aggregation and EB-like morphology, consistent with centrifugal force enhancing cell-cell attachment via induced cell shape change (Kinoshita et al., 2020). These force-dependent cellular events involve accumulation of F-actin at the apical cell surface and tight junction components (*Tjp1* or *ZO-1*) at the junction, enhancing cell-cell contact. Mechanical force can trigger Erk2 activation, remodelling cell-cell adhesion and tight junctions. It is also been reported that ZO-1 conformation changes in a tension-dependent manner (Spadaro et al., 2017). We have not studied the mechanisms underlying partial physical rescue of the *Grhl2-KO* phenotype by centrifugation. We speculate that the centrifugal force may have enhanced bridging of cell-cell junctions, possibly through accumulating junctional proteins, which would compensate for the loss of *Grhl2* and associated downregulation of junctional markers (*Cdh1 Cldn12*, *Ocln*, and *Tjp2*). However, spinning did not completely rescue KO morphology, indicating that the reduction of molecular mediators of cell adhesion, e.g., Cdh1, could not be overcome by mechanically enforcing cell-cell interactions. Instead, prolonging EB-like formation by a few days allowed the formation of more homogenous and compact aggregates.

Despite the initial differences in EB-like size and shape, and the carry-on effect seen up until about Day 7 of differentiation, KO cells developed along similar pathways to the wild-type cells. This indicates a change in developmental timing, rather than a complete loss of developmental ability.

Self-guided organogenesis within organoid differentiation protocols can be highly variable (Fatehullah et al., 2016; Huch et al., 2017). The IELO differentiation method used here relies on a homogenous, naïve population of ESCs forming a uniform, spherical aggregate composed of ∼3,000 cells. These were treated at specific time points to sequentially induce neural and non-neural ectoderm, eventually forming the otic placode and otic vesicles, respectively (Koehler et al., 2013; Koehler and Hashino, 2014). The differentiation protocol entails about 3 weeks of culture and results in heterogenous aggregates of <1 mm in diameter that contain multiple structures and cell types. The ability to accurately reproduce the IELO end-points relies on many factors: a homogenous ESC starting population; the specific ESC lines and strains used; complete incorporation of these cells into aggregates; uniform aggregate size and shape; batch-batch variation in reagents; timing for treatment with small molecules and proteins, which varies within a 24–48 h window for different cell lines (Eiraku et al., 2008; Eiraku and Sasai, 2011; Koehler et al., 2013). While differentiation of *Grhl2-KO* EB-like aggregates yielded similar structures to those produced by wild-type, the variability of the resulting organoids made quantification difficult. When a phenotype has already been described in other systems, for example, *in vivo* mouse studies, the IELO protocol can provide additional valuable insights. This was exemplified by analysing cellular phenotypes from mutations in the type II transmembrane protease 3 gene and its effect on hair cell apoptosis (Tang et al., 2019).

During bronchosphere organoid culture, loss of *GRHL2* after formation of apical junction complexes delocalised *TJP2*, and decreased levels of *CDH1* and *CLDN4* (Gao et al., 2013). This induced morphological changes towards less closely adherent cells with a more extended morphology. Single cells seeded into an ECM and cultured for 3 weeks had reduced clonogenicity, produced smaller spheres and lacked ciliated cells (Gao et al., 2013). Conditional *GRHL2* deletion of exons 1 or 3 was further analysed in mouse tracheal cells *in vivo* and in 3D tracheospheres (Gao et al., 2015). Again, *GRHL2* KO cells were less clonogenic and produced smaller organoids, indicative of reduced proliferation or decreased luminal spaces. The luminal cells within the spheres were flatter, more squamous with shorter basolateral membrane domain and fewer ciliated cells than wild-type controls. Wild-type cells compensated for the loss of conditionally deleted *GRHL2* in basal tracheal cells, maintaining epithelial barrier function and demonstrating the non-cell-autonomous actions of *GRHL2*. We observed a similar rescue of EB-like morphology in *Grhl2-KO* ESCs complemented with equal numbers of wild-type ESCs. By stimulating proliferation and organising epithelial integrity, *GRHL2* appears to play similar roles during airway and inner ear development.

## Data Availability

The original contributions presented in the study are included in the article/Supplementary Material, further inquiries can be directed to the corresponding author.

## Supplementary material

SUPPLEMENTARY FIGURE S1 Validating *gRNA/Cas9* genome edits of murine *Grhl2*. A commercial Grhl2 gRNA/Cas9 KO HDR kit (SCBT) was used, consisting of pooled plasmids: **(A)** *Grhl2* gRNA/Cas9 KO Plasmid (sc-434250), targeting exons 2 and 3 (red arrows—**(C)**, and **(B)** two HDR plasmids (sc-434250-HDR) with homology arms (pink) targeting exons 2 and 3. This plasmid combination was used to produce clonal ESC strains 3KO and 2KO. Gels show screening for integration of HDR (F^Plasmid^/R^HDR^) and KO (F^Plasmid^/402R) plasmid. **(C)** Murine *Grhl2* wild-type (WT) genomic DNA (gDNA) with untranslated regions (black) and coding sequence (CDS, grey) and complementary 5’ and 3’ homology arms (pink). Shown are binding sites for primers against exon 2 (primer pair: 2F/2R) and exon 3 (3F/3R). **(D)** Detection of edited exon 2 (2F/R^HDR^ and F^HDR^/2R) after homology-directed (HDR) insertion of red fluorescent protein (RFP) and puromycin (Puro) transgene. **(E)** Edited exon 3 (3F/R^HDR^ and F^HDR^/3R). **(F)** Edited exon 13 (primer pair: 13F/13R) after non-homologous end joining (NHEJ) showing Sanger sequencing. **(G)** Presence of *Grhl2* RNA with primers against exons 2-4 (2F^mRNA^/3R^mRNA^) and 10-16 (10F^mRNA^/16R^mRNA^). 3/2/1-KO = knockout of transactivation (blue), DNA-binding (yellow) and/or dimerisation (green) domains, respectively; M = marker; ntc = no template controls (water); Null = other clonal ESC strains that were not characterised further; RT- = no reverse transcriptase control.

SUPPLEMENTARY FIGURE S2 Reduced proliferation in *Grhl2–KO* ESCs and rescue of EB-like formation. **(A)** Proliferation rate of 2KO/3KO and 1KO compared to wild-type (WT) ESCs. Cells within colonies were counted five days after plating single ESCs (clonal culture) and populations of ESCs in standard 96-well and 96-well E-plates (group culture). **(B)** xCelligence real-time kinetic profiling. Normalised mean cell indices and standard deviation (light grey) were determined in 15 min intervals for 12 days. Sigmoid curves were fitted using the Growthcurver package in R (*n* = 2 biological replicates, N = 48 technical replicates per genotype). **(C)** Cell index quantification from xCelligence growth curve. **(D)** Representative immunoblot for proliferating cell nuclear antigen (PCNA) and reference histone 3 (H3) from group colonies. **(E)** Results represent normalised abundance for each genotype. *, **, *** = *p* < 0.05, *p* < 0.01, and *p* < 0.001, respectively, abc = groups with different letters differ by *P* < 0.01.

SUPPLEMENTARY FIGURE S3 Validating transgenic overexpression lines. **(A)** PCR from genomic DNA of *Grhl2-KO-Grhl1-TG* and *Grhl2-KO-Grhl2-TG* was amplified using primers specific for the *Grhl* ORFs. **(B)** *3KO-Grhl1-TG* and *3KO-Grhl2-TG* sequence confirmation of integration. ORF nucleotide sequences (3’ end only) with 100% identity to the reference sequence. Exon 16 (grey) of *Grhl1* and *Grhl2* ending in a histidine tag (HIS) and Not1 restriction enzyme (RE) cut site. Generated using Geneious Prime® 2019.1.1.

SUPPLEMENTARY FIGURE S4 Grhl2 is required for early IELO differentiation. **(A)** Representative images of single IELOs of different genotypes (WT, KO) tracked for eight days of differentiation (day 1–8). Scale bars = 400 µm. **(B)** Quantitative morphometry of area, perimeter, circularity and compactness during IELO differentiation. Data from SFEBq and SFEBq-spin culture conditions were pooled.
