# Supplementary material for: Grainyhead-like 2 is required for morphological integrity of mouse embryonic stem cells and orderly formation of inner ear-like organoids
Source: Front Cell Dev Biol. 2023 Sep 7;11:1112069. doi: 10.3389/fcell.2023.1112069 (PMC10513505; doi:10.3389/fcell.2023.1112069)
Supplement: Supplementary file 5 [file DataSheet1.docx]

**Supplemental Tables**

**Supplemental Table S1.** Sequences for genome editing tools. The PAM site of the gRNA is highlighted in red text.

| **Name** | **Sequence (5’‑3’)** |
| --- | --- |
| gRNA‑125F | CACCGAAAGAAGAAAACGTAAAACGAGG |
| gRNA‑125R | AAACCCTCGTTTTACGTTTTCTTCTTT |
| gRNA‑124F | CACCGTTGATCTGCTTAGACGGTGTTGG |
| gRNA-124R | AAACCCAACACCGTCTAAGCAGATCAA |
| Exon 13 sequencing 140F | TTCATCTCTGTGTGTCCTCT |
| Exon 13 sequencing 130R | GAGTGAGTTCTTCACAG |
| Exon 2 5’ Homology arm | AGAGAGATTCTGATTCACCTAGAACTGTAGTTACAGACAGTTGTAACTACAGTTATCTGATGTGAGGGCTGGGAATTGAACCTGGTCCTCTGGAAATGCAGTAAGCACTCTTAACCACTGAGCCATTTCTTCAAGTCCAGGCTACCTTTTAAAAGCTATTACGACTCTTCATACACAGTGAAGGTGTGAGTTGAGGCTTTTAAAGTGCAGTGTGGTGGTCATTTCAGCTTTAACATGCCTTAATCTGGGCAAATACAAATCAGGGTAACCACGTGACATGTATGGTATCAGTGGAAAAACATGAAAAGTTCCTGGGCAGTGAGCCTTACATCCCTCTTGTGTTTTACAGTAATAAAAGACTAGTGGCCTTAGTGCCCATGCCCAGTGACCCTCCCTTCAACACCCGAAGAGCCTACACAA |
| Exon 2 3’ Homology arm | CTGGAAGTCATATCTGGAGAACCCCCTGACTGCGGCCACCAAGGCGATGATGAGCATCAACGGGGACGAGGACAGTGCTGCCGCCCTGGGCCTGCTCTATGACTACTACAAGGTGGGTCTCTCCCTTGGGCCAGCCCCTCCTCCCTGCCTCCAGTGGCAAGACAGAACAGTCTTTGTTCTAGGTCAGTGGCCTTCCTTAGGTGCTTGGTTTTGGTTCCCCCTCCCCCCTTGTGAAACCCAACTTCATGTCCAGAGAAGTTGTAATTTGGGGATTCTCTTTGTCTCCGTCTCTGTCTGCCTCTCGAGTGTCTGCCTCTCATGTGTGTGTGTGTCTGTGTGCAGAGGCCACAAGAGGACGGCTGGCGGATCCCCAGGAGTTGGAGTCTTACCTCCTTTCTGTCATCTTACTTCGTGGTTAGGAGCTGGCTTTATGTGGCACTGCCATGACAGTTCAGCTGTCAACCTTTACTTGTGTC |
| Exon 3 5’ Homology arm | ATCTATACATCTGTAGGTAGTGGAGTCTCCTCTCTTCCACAAGGACTTTTCAATATAATATGTCAGGTGGCATCATGCTGGCATCATGGTTAGAAATCAACTGCATCAGAAAGACTGGAGGCAAGCTTCCTAGGGAGTGCTTAGGGAATGTCCCTAGTGGATGCTCAGAGTCAAAGGAGGAACTGTCAGGCCCTGCCCAGTGTCTCCCTAGCCCTCATCAGCCTGGCGCCAAGCTGCAGATAGGCAGCTTCCTCTGTGCTCCCAATGGGTTGCTCTCTCTCAAGCCCACCTTCACCCCACACACTTGGCTCTTCCCCTCACCCATTTCTACATCTGGGAGCACACAGCATTGTTTTCTATGTCAACCATGGTGATCATGAACCTACACTGCTCGCCGCTTTCTCGGGTTCCTCTTAACCATTTCCTGATAATAACCTTCTACTTCCAAAAAAAAAAGTTCCCGTTAGTTTCTTTGCCTGTGTTGGTATCTCCATCTGCACACGGTCAGTAAGTCTGTCTGATGGTTTACTGCTGGTT |
| Exon 3 3’ Homology arm | TTCAGGTTCCTCGAGACAAGAGACTTCTGTCTGTGAGCAAAGCAAGTGACAGCCAAGAAGACCAGGATAAAAGGTACAATGAGAGGGAGTCTGTGCGCCTCCTAGAAAAAGTCTTTTTCTATGGGTTTTGAAAAAAGGAAGCCAGGCACAATGGTATATGACTTTGTTGCCAGCACTTAGGAAGCAGAGGCAGGTGGATCTCGGTGGGTTTCAGGTCAACCTGATCTACGTATTAAATTCCAAGCAGCCAGGGCTACATAGAGAGACCTTGTCTCAAAAACCTATCCATCCCCAACATGACCTGAAGAGTGGCTTACCAGTTTAGTTGCTGTTGGTCATGACCTGGGTTTCTTTCCCAGTCCCCACATGGTGGTTCACAACCTTCCTAACTCCAGTTTCAAGGGATCTGTTACCCTATTTCGATGTCCAAGGGGACTGGACATGCAAGTGGTCCACATAGACACAGTCAGGCAAAAGCATCTACACATAAAGTTAAAATATAGCTTGAGCTGGAAGTTTGTTTTTAGAAGAGGTAGGGCCAAGAAGCCTCACTTCTTCTGTCTTAGGAGCCTGGACCAAAACTTGTTCTCTTGCTAGGAAAAGCAAATGACATATTTTTGCCTGTGCATCCAGAGGGTCTTCAGCAAGTTTCCTCAGATGAAGAGCTGATGAGACAGTTAAAACTCAAGAGTTAGAGGTGATTGCCAGTAAGAGCCTGCTGCTGAACGCAGCTTTCTGCCCAGCTCCACACTCTTTCCTGCAGTCTGGCCCTTAAGGGAATGATTGCTCTTAGT |

**Supplemental Table S2.** Primers pairs for confirming gene editing and transgene overexpression.

| **Name** | **Size (bp)** | **Primer** | **Sequence (5’- 3’)** |
| --- | --- | --- | --- |
| *Grhl2 – exon 2* | 572 | 2F | AAAGTGCAGTGTGGTGGTCA |
|  |  | 2R | GCACACAGACACACACACAC |
| *Grhl2 – exon 3* | 1981 | 3F | CTCAGACGCCAGCTTTCTCA |
|  |  | 3R | AGAGCAGAGACTCGATGGGT |
| *Grhl2 – exon 3* | 330 | 3aF | CGCTTTCTCGGGTTCCTCTT |
|  |  | 3aR | TCTGCTTCCTAAGTGCTGGC |
| *Grhl2/HDR – exon 2* | 565 | 2F | AAAGTGCAGTGTGGTGGTCA |
|  |  | R^HDR^ | CCCGTTGCGAAAAAGAACGT |
| *HDR/Grhl2 – exon 2* | 563 | F^HDR^ | CCTTAGGCCTCCTCCTTCCT |
|  |  | 2R | GCACACAGACACACACACAC |
| *Grhl2/HDR – exon 3* | 489 | 3aF | CGCTTTCTCGGGTTCCTCTT |
|  |  | R^HDR^ | CCCGTTGCGAAAAAGAACGT |
| *HDR/Grhl2 – exon 3* | 248 | F^HDR^ | CCTTAGGCCTCCTCCTTCCT |
|  |  | 3aR | TCTGCTTCCTAAGTGCTGGC |
| *Grhl2/NHEJ – exon 13* | 767 | 13F | CAGCAGCGTCCTTGTTAAGC |
|  |  | 13R | TCAGAGTGCCAAGCAAGGAG |
| *HDR plasmid* | 2146 | F^Plasmid^ | CAGACAAGCTGTGACCGTCT |
|  |  | R^HDR^ | CCCGTTGCGAAAAAGAACGT |
| *KO plasmid* | 1912 | F^Plasmid^ | CAGACAAGCTGTGACCGTCT |
|  |  | R^GFP^ | GAGTACAGGTGTGCAGCTCT |
| *Grhl2 mRNA – Exon 2-4* | 189 | 2F^mRNA^ | GAAGAGCCTACACAAGTGAG |
|  |  | 3R^mRNA^ | GGCTGTCACTTGCTTTGCTC |
| *Grhl2 mRNA – Exon 14-16* | 196 | 53F | GAAGAGCCTACACAAGTGAG |
|  |  | 53R | GGCTGTCACTTGCTTTGCTC |
| *Grhl2 mRNA – Exon 10-16* | 182 | 11F^mRNA^ | CCCAGTGCAACAACTCCTCT |
|  |  | 16R^mRNA^ | TCGCTCATCGTCTGTGTTGT |
| *Grhl2 mRNA – Exon 10-16* | 554 | 10F^mRNA^ | CCCAGTGCAACAACTCCTCT |
|  |  | 53R | GGGCTCAGATCTCCATCAGC |
| *Grhl2 mRNA – Exon 10-12* | 182 | 10F^mRNA^ | CCCAGTGCAACAACTCCTCT |
|  |  | 12R^mRNA^ | TCGCTCATCGTCTGTGTTGT |
| *Grhl1 expression plasmid* | 1774 | 29F | TGGAAAACCCTCTCACTGCG |
|  |  | 765R | GGAGGTGTGGGAGGTTTTT |
| *Grhl2 expression plasmid* | 1638 | 3F^mRNA^ | GCTTCCTCTGTGCTCCCAAT |
|  |  | 765R | GGAGGTGTGGGAGGTTTTT |

**Supplemental Table S3.** Primer pairs used for RT-qPCR.

| **Gene** | **Size (bp)** | **Sequence (5’- 3’)** | | **Mean  reaction efficiency** | **Reference** |
| --- | --- | --- | --- | --- | --- |
| *Actb* | 200 | F | CAGAAGGACTCCTATGTGGG | 1.99986 | [1] |
|  |  | R | TTGGCCTTAGGGTTCAGGG |  |  |
| *Gapdh* | 176 | F | CCCACTAACATCAAATGGGG | 1.99988 | [2] |
|  |  | R | CCTTCCACAATGCCAAAGTT |  |  |
| *Gusb* | 96 | F | ATAAGACGCATCAGAAGCCG | 1.99987 | [1] |
|  |  | R | ACTCCTCACTGAACATGCGA |  |  |
| *HPRT* | 332 | F | GAAATGTCAGTTGCTGCGTC | 1.99993 | [1] |
|  |  | R | GCCAACACTGCTGAAACATG |  |  |
| *Cdh1* | 181 | F | GCTGGACCGAGAGAGTTACC | 1.99989 | This paper |
|  |  | R | CCGGGCATTGACCTCATTCT |  |  |
| *Cdh2* | 191 | F | CCTTGCTTCAGGCGTCTGTG | 1.90245 | This paper |
|  |  | R | CTTGAAATCTGCTGGCTCGC |  |  |
| *Cldn12* | 191 | F | CTGATCCCCTTTCCCTGTGC | 1.98704 | This paper |
|  |  | R | GGCTTCGCCAGAACGCA |  |  |
| *Grhl1* | 177 | F | TGGAAAACCCTCTCACTGCG | 1.99991 | This paper |
|  |  | R | TGCTGTTTCTTTTGCTGTGCT |  |  |
| *Grhl2* | 182 | F | CCCAGTGCAACAACTCCTCT | 1.99985 | This paper |
|  |  | R | TCGCTCATCGTCTGTGTTGT |  |  |
| *Grhl3* | 184 | F | AGCCAAGGAAGATGACCTTCAG | 1.99994 | This paper |
|  |  | R | CAGGATGCCTCGCTTGCATT |  |  |
| *Myo7a* | 195 | F | CAGATCCAGGTGGTGGATGA | 1.903178 | This paper |
|  |  | R | ACCTGTGTACGTATAGATGAGGT |  |  |
| *Nanog* | 223 | F | CAGGTGTTTGAGGGTAGCTC | 1.89279 | [2] |
|  |  | R | CGGTTCATCATGGTACAGTC |  |  |
| *Nes* | 187 | F | GCAGGAGAAGCAGGGTCTAC | 1.865909 | This paper |
|  |  | R | CTTGGGGTCAGGAAAGCCAA |  |  |
| *Ocln* | 181 | F | GCCGCCAAGGTTCGCT | 1.99985 | This paper |
|  |  | R | GACATGCATCTCTCCGCCAT |  |  |
| *Otx2* | 199 | F | CACCTCTACTTTGATAGCTGGC | 1.897739 | This paper |
|  |  | R | GTTGTTTGGAGGCGCAAAGT |  |  |
| *Pou5f1* | 492 | F | CCTGGAATCGGACCAGGCTCAGAGGTATTG | 1.89763 | [1] |
|  |  | R | ATTGTTGTCGGCTTCCTCCACCCACTTCTC |  |  |
| *Sox2* | 277 | F | AGCTACGCGCACATGAACGGCTGGAGCAAC | 1.91883 | [1] |
|  |  | R | TGGAGCTGGCCTCGGACTTGACCACAGAGC |  |  |
| *Tjp1* | 181 | F | GCCGCCAAGGTTCGCT | 1.99986 | This paper |
|  |  | R | GACATGCATCTCTCCGCCAT |  |  |
| *Tjp2* | 183 | F | TCCACTGCAGCTTGTAGTTCT | 1.99926 | This paper |
|  |  | R | TTTGGAATCCTTCTGCAGGGTC |  |  |
| *Tubb3* | 170 | F | TGAGGCCTCCTCTCACAAGT | 1.93861 | This paper |
|  |  | R | CCTCCGTATAGTGCCCTTTGG |  |  |

**Supplemental Table S4.** Primary antibodies used for immunofluorescence (IF) or western blot (WB).

| **Antigen** | **Reactivity** | **Company** | **Catalog No.** | **IF/WB** |
| --- | --- | --- | --- | --- |
| Cdh2 | Mouse | Santa Cruz | sc‑7939 | 1:50 |
| H3 | Rabbit | Abcam | ab1791 | 1:10000 |
| PCNA | Mouse | Abcam | ab29 | 1:2000 |

**Supplemental Table S5.** Secondary antibodies used for immunofluorescence (IF) and western blot (WB).

| **Host** | **Reactivity** | **Conjugate** | **Company** | **Catalog No.** | **IF/WB** |
| --- | --- | --- | --- | --- | --- |
| Donkey | Mouse | Alexa-Fluor 488 | ThermoFisher | A‑21202 | 1:2000 |
| Goat | Rabbit | HRP | Dako | PO448 | 1:10000 |
| Donkey | Mouse | Alexa-Fluor 568 | ThermoFisher | A 10037 | 1:10000 |

**Supplemental References**

[1] J. Antony, F. Oback, L.W. Chamley, B. Oback, and G. Laible, Transient JMJD2B-mediated reduction of H3K9me3 levels improves reprogramming of embryonic stem cells into cloned embryos. Molecular Biology of the Cell 33 (2013) 974-83.

[2] J. Silva, J. Nichols, T.W. Theunissen, G. Guo, A.L. van Oosten, O. Barrandon, J. Wray, S. Yamanaka, I. Chambers, and A. Smith, Nanog is the gateway to the pluripotent ground state. Cell 138 (2009) 722-37.

**Supplemental Figure Legends**

**Supplemental Figure S1.** Validating *gRNA/Cas9* genome edits of murine *Grhl2*. A commercial

Grhl2 gRNA/Cas9 KO HDR kit (SCBT) was used, consisting of pooled plasmids: (A) Grhl2 gRNA/Cas9 KO Plasmid (sc-434250), targeting exons 2 and 3 (red arrows—(C), and (B) two HDR plasmids (sc-434250-HDR) with homology arms (pink) targeting exons 2 and 3. This plasmid combination was used to produce clonal ESC strains 3KO and 2KO. Gels show screening for integration of HDR (F^Plasmid^/R^HDR^) and KO (F^Plasmid^/402R) plasmid. (C) Murine *Grhl2* wild-type (WT) genomic DNA (gDNA) with untranslated regions (black) and coding sequence (CDS, grey) and complementary 5’ and 3’ homology arms (pink). Shown are binding sites for primers against exon 2 (primer pair: 2F/2R) and exon 3 (3F/3R). (D) Detection of edited exon 2 (2F/R^HDR^ and F^HDR^/2R) after homology-directed (HDR) insertion of red fluorescent protein (RFP) and puromycin (Puro) transgene. (E) Edited exon 3 (3F/R^HDR^ and F^HDR^/3R). (F) Edited exon 13 (primer pair: 13F/13R) after nonhomologous end joining (NHEJ) showing Sanger sequencing. (G) Presence of *Grhl2* RNA with primers against exons 2-4 (2F^mRNA^/3R^mRNA^) and 10-16 (10F^mRNA^/16R^mRNA^). 3/2/1-KO = knockout of transactivation (blue), DNA-binding (yellow) and/or dimerisation (green) domains, respectively; M = marker; ntc = no template controls (water); Null = other clonal ESC strains that were not characterised further; RT- = no reverse transcriptase control.

**Supplemental Figure S2.** Reduced proliferation in *Grhl2–KO* ESCs and rescue of EB-like formation. (A) Proliferation rate of 2KO/3KO and 1KO compared to wild-type (WT) ESCs. Cells within colonies were counted five days after plating single ESCs (clonal culture) and populations of ESCs in standard 96-well and 96-well E-plates (group culture). (B) xCelligence real-time kinetic profiling. Normalised mean cell indices and standard deviation (light grey) were determined in 15 min intervals for 12 days. Sigmoid curves were fitted using the Growthcurver package in R (n = 2 biological replicates, N = 48 technical replicates per genotype). (C) Cell index quantification from xCelligence growth curve. (D) Representative immunoblot for proliferating cell nuclear antigen (PCNA) and reference histone 3 (H3) from group colonies. (E) Results represent normalised abundance for each genotype. *, **, *** = p < 0.05, p < 0.01, and p < 0.001, respectively, abc = groups with different letters differ by P < 0.01.

**Supplemental Figure S3.** Validating transgenic overexpression lines. (A) PCR from genomic DNA of *Grhl2-KO-Grhl1-TG* and *Grhl2-KO-Grhl2-TG* was amplified using primers specific for the *Grhl* ORFs. (B) *3KO-Grhl1-TG* and *3KO-Grhl2-TG* sequence confirmation of integration. ORF nucleotide sequences (3’ end only) with 100% identity to the reference sequence. Exon 16 (grey) of *Grhl1* and *Grhl2* ending in a histidine tag (HIS) and Not1 restriction enzyme (RE) cut site. Generated using Geneious Prime® 2019.1.1.

**Supplemental Figure S4.** Grhl2 is required for early IELO differentiation. (A) Representative images of single IELOs of different genotypes (WT, KO) tracked for eight days of differentiation (day 1–8). Scale bars = 400 μm. (B) Quantitative morphometry of area, perimeter, circularity and compactness during IELO differentiation. Data from SFEBq and SFEBq-spin culture conditions were pooled.
